# Utility of Infrared Thermography in Assessing Synovitis in Rheumatoid Arthritis: An Observational Study

**DOI:** 10.7759/cureus.101573

**Published:** 2026-01-14

**Authors:** Nidhish M Chandra, Puneet Kumar, Urmila Dhakad, Shweta Bhardwaj, Sanjay Buddha Pruthviraj, Parag Vijayvergia, Lily Singh

**Affiliations:** 1 Clinical Immunology and Rheumatology, King George's Medical University, Lucknow, IND

**Keywords:** disease activity score (das28-esr), infrared thermography, joints, mcp joints, metacarpophalangeal joints, pip joints, proximal interphalangeal joints, rheumatoid arthritis, synovitis, tenosynovitis

## Abstract

Introduction

There is an unmet need for an objective, cost-effective, and user-friendly tool to assess disease activity in rheumatoid arthritis (RA). This pilot study explores the utility of infrared thermography (IRT) in evaluating temperature differences between diseased and normal joints, and its correlation with the Disease Activity Score in 28 joints using Erythrocyte Sedimentation Rate (DAS28-ESR).

Methods

We recruited 50 RA patients (90% female subjects, mean age 47.7 years, mean disease duration 6.2 years) and 50 age- and sex-matched healthy controls. Clinical disease activity was assessed using DAS28-ESR, and IRT was performed across 28 joints, with mean joint temperature (T mean) compared between groups and correlated with disease activity.

Results

Of the 1400 diseased joints assessed, 126 were swollen and 297 were tender, with 21 patients (42%) showing high disease activity (mean DAS28-ESR 4.59). T mean was significantly higher in patients with RA than controls across all 28 joints (p<0.05), with a moderate correlation to DAS28-ESR (r=0.646). Receiver Operating Characteristic (ROC) analysis yielded an area under the curve (AUC) of 0.84, with 83% sensitivity and 73% specificity at a T mean cut-off of 33.45°C. Thermography detected tenosynovitis in two patients, subclinical synovitis in eight patients in clinical remission, and differentiated active from damaged joints in 10 patients.

Conclusion

IRT is a promising, non-invasive tool for detecting joint inflammation in RA, with the potential to enhance everyday clinical practice by identifying subclinical synovitis, tenosynovitis, and active joints, particularly in resource-limited settings.

## Introduction

Rheumatoid Arthritis (RA) is an inflammatory systemic autoimmune disorder primarily affecting joint synovium with a global prevalence of 0.24% and contributing up to 4% of all musculoskeletal pain cases [1,2]. The recent advances in the field of RA have made it very important to assess disease activity for optimum treatment response. Clinical examination, musculoskeletal ultrasound, and magnetic resonance imaging (MRI) are the only validated tools in assessing disease activity in RA, but these are time-consuming, costly, and require skilled professionals [3,4]. Hence, there is an unmet need for a cost-effective, easy-to-use, and precise tool to assess disease activity in RA. 

Infrared thermography (IRT) is a noninvasive modality that uses emitted infrared radiation produced by heat to see alterations in the surface temperature of an object. As inflammation produces heat, thermography has the potential to detect joint inflammation [5]. Modern infrared cameras are compact and portable, allowing rapid image acquisition over a very short time span, which makes it more convenient than other modalities like MRI and ultrasonography [6]. Thermal imaging has been previously studied in the medical arena for various conditions like diagnosing breast cancer [7-10], detecting early stages of diabetic neuropathy [11], mass screening of infectious diseases [12,13], monitoring peripheral blood flow [14], diagnosing and monitoring rheumatic diseases [15-18], detecting liver metastasis, and monitoring liver functioning [19].

Joint inflammation is the cardinal feature of RA. As early as the first century A.D., the Roman encyclopedist Celsus recognized and recorded that “calor” or heat is one of the four classic signs of inflammation [20]. Frize et al. [21] and Snekhalatha et al. [22] performed passive thermographic studies on patients with RA and found increased temperature when compared with healthy participants. Borojevi et al. [23] collected thermographic images of hands in patients with RA, osteoarthritis, and those of normal subjects and found that the heat distribution in all the three categories was different. Though these studies paved the way for further research in utilizing thermal imaging for assessing joint inflammation, most of the clinicians are still unaware of such a modality. Hence, this study aims to assess the utility of IRT in detecting synovitis in patients with RA and compare the thermography findings with the clinical gold standard.

Study objective

The primary objective of this pilot study was to evaluate the utility of IRT using a handheld cost-effective compact thermal camera in detecting joint temperature elevations in patients with RA compared to healthy controls, and to assess its correlation with clinical disease activity via the Disease Activity Score in 28 joints using Erythrocyte Sedimentation Rate (DAS28-ESR).

## Materials and methods

This observational pilot study was conducted from April 2023 to April 2024 after obtaining institutional ethics committee approval (approval number: XV-PGTS-IIA/P35). Fifty adult patients (>18 years) diagnosed with RA according to the 2010 American College of Rheumatology/European League Against Rheumatism (ACR/EULAR) classification criteria [24] and 50 age- and sex-matched healthy controls were enrolled after obtaining written informed consent. Patients with recent joint trauma, intra-articular metallic implants, or pregnancy were excluded.

Clinical disease activity was assessed using the DAS28-ESR score [25], which was used to classify patients into remission (DAS28-ESR < 2.6), low activity (2.6 ≤ DAS28-ESR ≤ 3.2), moderate activity (3.2 < DAS28-ESR ≤ 5.1), or high disease activity (DAS28-ESR > 5.1). IRT was performed across the 28 joints included in the DAS28 score, and ultrasonography was conducted for joints with discordant clinical and thermographic findings. Clinical examinations, thermographic assessments, and ultrasonography were done by three separate clinicians who were blinded to each other's findings to minimize bias.

Statistical analysis

To evaluate the diagnostic performance of IRT in detecting clinical disease activity, a Receiver Operating Characteristic (ROC) analysis was performed. The mean joint temperature (T mean) across the 28 joints was used as the test variable, with clinical disease activity (defined as DAS28-ESR >3.2 for moderate/high activity vs. ≤3.2 for remission/low activity) as the reference standard. The area under the ROC curve (AUC) was calculated to assess discriminatory ability, with sensitivity and specificity derived at the optimal T mean cut-off determined by the Youden index.

IRT procedure

The study was performed using a compact thermal camera FLIR C5 (Teledyne FLIR, Teledyne Technologies, Oregon, US), which had a resolution of 19,200 pixels and an infrared sensor of 160*120 with thermal sensitivity of <70 mK. The cost of the camera was around 900 USD (75,000 INR). To help patients acclimatize as per standard practice, they were required to arrive at the study premises at least 15 minutes prior to imaging and were made to sit comfortably in a room that was designated for the study. Assessment of all the 28 joints by IRT for each patient took around 15 minutes (both wrists, metacarpophalangeal (MCP) and proximal interphalangeal (PIP) joints were captured in a single shot). Hence the total evaluation time for each patient was 30 minutes (15 minutes for getting acclimatized and 15 minutes for image acquisition). The images were analyzed using FLIR tools software for Regions of Interest (ROI) placement and temperature extraction. 

Room settings

The study room was maintained at a controlled temperature of 25°C, without windows, to minimize environmental interference. Participants exposed their limbs up to the shoulders and knees, and removed their shoes and socks during the procedure. The thermal camera was positioned 50 cm perpendicular to the patient’s skin surface, with joints placed in a neutral position on a flat tabletop. For hand imaging, the dorsal view was captured to align with the anterior joint profiles. ROIs were selected using a standardized protocol: a circular ROI (fixed 5x5 cm²) was manually placed over each anatomical site (wrists, MCPs, interphalangeal joints (IPs), PIPs, elbows, shoulders, and knees) using anatomical landmarks. ROI placement was verified by two trained operators to ensure consistency. Thermographic parameters analyzed included maximum (Tmax), minimum (Tmin), and average (Tavg) temperatures, consistent with prior studies. No formal inter/intra observer variability was assessed in this pilot study. 

## Results

The baseline characteristics of the patients are depicted in Table 1.

**Table 1:** Baseline characteristics of the study population DAS28-ESR: Disease Activity Score in 28 joints using the Erythrocyte Sedimentation Rate.

Parameter	Summary
Mean age in years (Standard Deviation or SD)	47.7 (SD 10.2)
Female gender	45 (90%)
Mean duration of illness (years)	6.2 (SD 4.1)
Total number of swollen joints examined	126
Total number of tender joints examined	297
Mean number of swollen joints per patient	2.52 (SD 1.8)
Mean number of tender joints per patient	5.94 (SD 3.2)
Mean patient global assessment score (PGA)	5.29 (SD 2.29)
Mean ESR (mm/first hour)	44.08 (SD 22.04)
Mean DAS28-ESR	4.59 (SD 1.66)

Out of the 50 patients included in the study, 45 (90%) were female subjects. The mean duration of the illness in the study population was 6.2 years (Standard Deviation or SD 4.1). As a part of the study, a total of 1400 diseased joints and 1400 normal joints were assessed. Out of the 1400 diseased joints, 126 were swollen, and 297 were tender clinically.

Of the 50 patients, 21 (42%) had high disease activity, 14 (28%) had moderate disease activity, 10 (20%) had low disease activity, and five (10%) were in remission based on their DAS28-ESR scores. IRT was done in 50 patients with RA and 50 age- and sex-matched controls. T mean was calculated from 28 joints included in the DAS28 score. Table 2 shows the T mean of 28 joints among the RA and control groups with their corresponding p values.

**Table 2:** Mean temperature (T mean) of 28 joints among the RA and control groups with their corresponding p values RA: rheumatoid arthritis; PIP: proximal interphalangeal; MCP: metacarpophalangeal. All T mean values are given in degree Celsius along with its standard deviation in brackets.

Joints	Right (RA)	Right (control)	P value	Left (RA)	Left (control)	P value
1^st^ PIP	33.75 (1.16)	32.15 (0.59)	0.00010	33.59 (1.11)	32.19 (0.60)	0.00031
2^nd^ PIP	33.60 (1.19)	32.67 (0.42)	0.0105	33.52 (1.17)	32.28 (0.49)	0.00113
3^rd^ PIP	33.95 (1.22)	32.93 (0.78)	0.0342	33.62 (1.17)	32.77 (0.59)	0.0224
4th PIP	33.68 (1.21)	32.17 (0.79)	0.00059	33.51 (1.15)	32.19 (0.70)	0.00110
5^th^ PIP	33.55 (1.11)	31.78 (0.49)	0.00001	33.53 (1.14)	31.69 (0.45)	0.00007
1^st^ MCP	33.69 (1.15)	32.40 (0.46)	0.00061	33.58 (1.09)	32.25 (0.64)	0.00055
2^nd^ MCP	33.57 (1.20)	31.81 (0.50)	0.00003	33.52 (1.18)	32.48 (0.75)	0.00990
3^rd^ MCP	33.61 (1.21)	32.29 (0.48)	0.00080	33.59 (1.10)	32.48 (0.65)	0.00318
4^th^ MCP	33.57 (1.15)	31.30 (0.48)	0.00003	33.57 (1.12)	31.73 (0.54)	0.000008
5^th^ MCP	33.55 (1.13)	32.75 (0.65)	0.0300	33.47 (1.11)	32.55 (0.76)	0.0167
Wrist	34.40 (1.20)	32.40 (0.47)	0.000004	34.27 (1.18)	32.38 (0.50)	0.000009
Elbow	33.88 (1.04)	31.86 (0.78)	0.000004	33.95 (1.07)	32.26 (0.57)	0.00002
Shoulder	33.72 (0.96)	32.95 (0.87)	0.0351	33.73 (0.94)	32.87 (1.1)	0.0351
Knee	34.53 (1.00)	32.35 (0.56)	0.000001	34.35 (1.04)	31.87 (0.67)	0.000006

These p-values were obtained using a t-test for independent samples, which helped determine the significance of the differences observed. There was significant statistical difference among both the groups in all the 28 joints with a p value of <0.05. Maximum temperature difference was observed in bilateral wrists, elbows, and shoulders.

**Figure 1:**
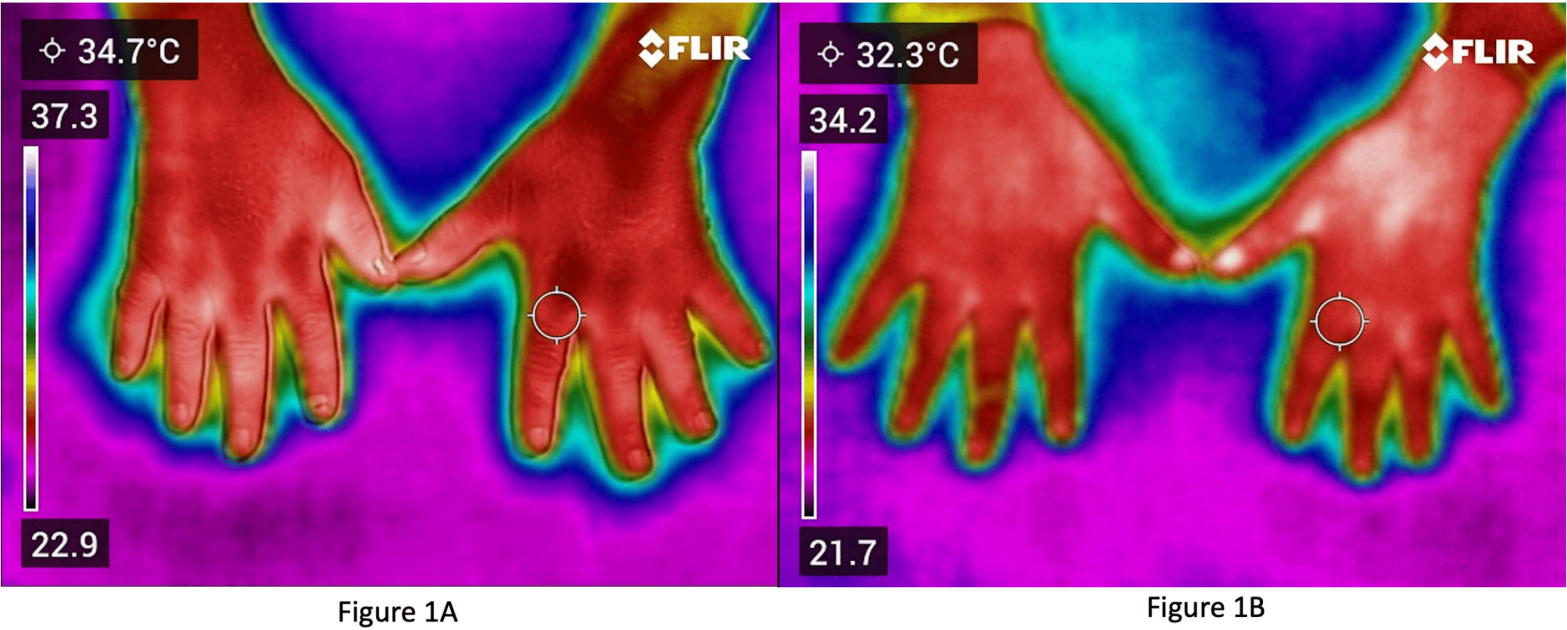
Infrared thermography images 1A: Shows the infrared thermography image of a patient with RA; 1B: Shows the infrared thermography in a normal subject.

Figure 1A shows the IRT image of a patient with RA.

This was a patient with clinically active joints who had a high disease activity as per the DAS28-ESR score. The circular box shows the temperature of 34.7°C over the second left MCP joint which is high as compared to a healthy subject. The figure also shows the temperature difference between the right and left hand in which the right-sided joints demonstrate higher temperature. The right hand shows areas of white color in the MCP and PIP joints indicating high temperature, whereas the left hand shows red areas which correspond to lower temperatures than the white areas in the right hand (blue stands for lowest temperature, red for high, and white for highest temperature).

Figure 1B shows IRT in a normal subject. The left second MCP joint has a temperature of 32.3°C. In a similar manner, the temperature of all the 28 joints was documented, compared with the RA group, and the p value was calculated.

Table 3 provides an overview of the T mean values corresponding to different levels of disease activity, as measured by DAS28-ESR.

**Table 3:** Mean temperature (T mean) for disease activity based on DAS28-ESR DAS28-ESR: Disease Activity Score in 28 joints using Erythrocyte Sedimentation Rate

Disease activity	T mean (°C)
Remission (SD – Standard Deviation)	32.8 (SD 0.4)
Low disease activity	32.92 (SD 0.6)
Moderate disease activity	33.7 (SD 0.7)
High disease activity	34.5 (SD 0.8)

The data was categorized into four distinct disease activity levels: (1) Remission: The mean temperature for patients in remission is 32.8°C. This indicates a relatively lower temperature associated with minimal disease activity; (2) Low disease activity: Patients experiencing low disease activity have a slightly higher mean temperature of 32.92°C. This small increase in temperature suggests a mild elevation in disease activity compared to remission; (3) Moderate disease activity: As disease activity progresses to a moderate level, the mean temperature rises to 33.7°C. This reflects a more noticeable increase in temperature, aligning with the increased inflammatory response associated with moderate disease activity; (4) High disease activity: For patients with high disease activity, the mean temperature reaches 34.5°C. This is the highest temperature recorded among the categories, indicating a significant inflammatory response and elevated disease activity.

The trend observed in the data suggests a positive correlation between T mean and disease activity levels, with higher temperatures corresponding to increased disease activity. Pearson's correlation coefficient was used to determine this correlation and it was 0.646 between DAS28-ESR and T mean. This indicates a moderate positive correlation between the two variables, suggesting that as one variable increases, the other tends to increase as well.

**Figure 2:**
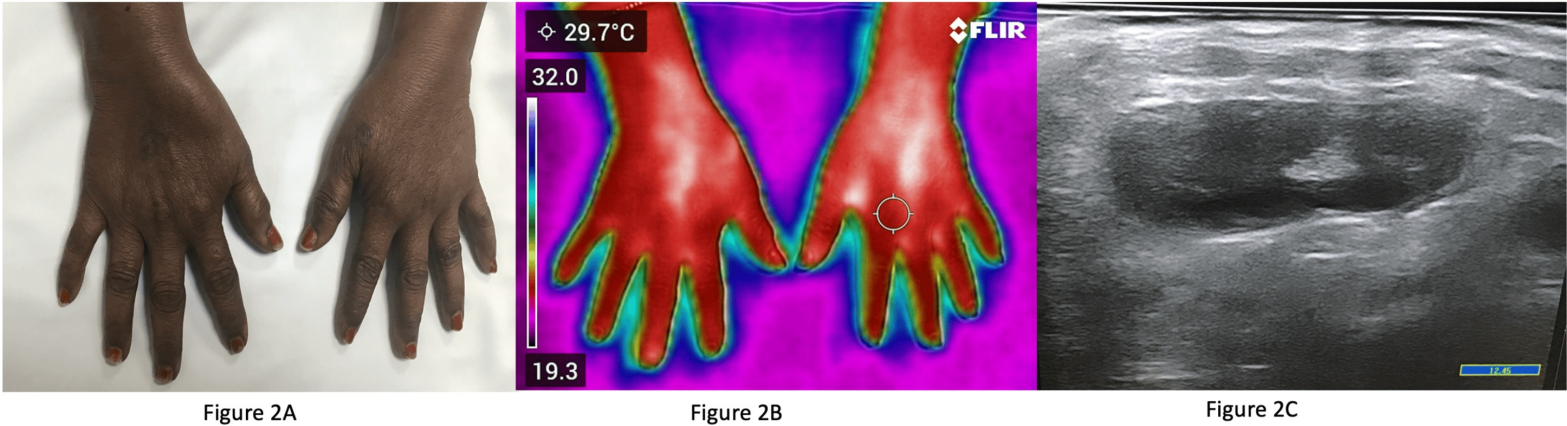
Image of a patient's hands 2A: Clinical image of a patient with swelling over the bilateral wrists extending across the joint line involving the extensor tendons; 2B: Infrared thermography of the same patient demonstrating increased temperature, which is distributed across the extensor tendon; 2C: Ultrasonography of the same confirming tenosynovitis.

Out of the 50 patients with RA, two patients had tenosynovitis at the extensor compartment of the wrist. Figure 2A shows the clinical image of a patient with swelling over the bilateral wrists extending across the joint line involving the extensor tendons.

IRT of this area showed increased temperature which was distributed across the extensor tendon (Figure 2B) unlike in active arthritis where it is localized to the joint. This tenosynovitis was confirmed by ultrasonography of the wrist (Figure 2C). 

Out of the 126 swollen joints examined, 101 joints showed increased temperature, correlating with the inflammation, whereas 15 joints had normal temperature. Ultrasonography of these joints didn’t demonstrate any synovitis, whereas X-ray of these joints showed evidence of damage. The other 10 joints had synovitis on ultrasound but IRT could not demonstrate increased temperature.

**Figure 3:**
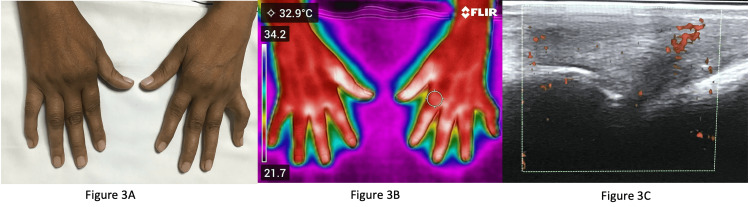
Image of a patient, whose hands were affected with rheumatoid arthritis (RA) 3A: Clinical image of a patient with long-standing RA; 3B: Infrared thermography of the same patient showing increased temperature over the right and left first, second, and third metacarpophalangeal (MCP) and proximal interphalangeal (PIP) joints; 3C: Ultrasonography findings of the same patient showing synovitis with power doppler uptake at the MCP joint.

Figure 3A shows the clinical image of a patient with long-standing disease, with normal inflammatory markers.

This patient had early morning stiffness of around 45 minutes and was on daily non-steroidal anti-inflammatory drugs (NSAIDs). Figure 3A shows flexion deformities at the left fifth PIP and right fifth PIP, volar subluxation of the right first, second, and third MCP joints. Clinically, this patient was thought to have damage rather than active disease. Figure 3B shows IRT of the same patient with an increased temperature over the right and left first, second, and third MCP and PIP joints. Figure 3C shows the ultrasonography findings of the same patient, exhibiting synovitis with power doppler uptake at the MCP joint. These findings are suggestive of active synovitis. Thus, thermography could detect areas of increased temperature which correlated with power doppler uptake in ultrasonography.

**Figure 4:**
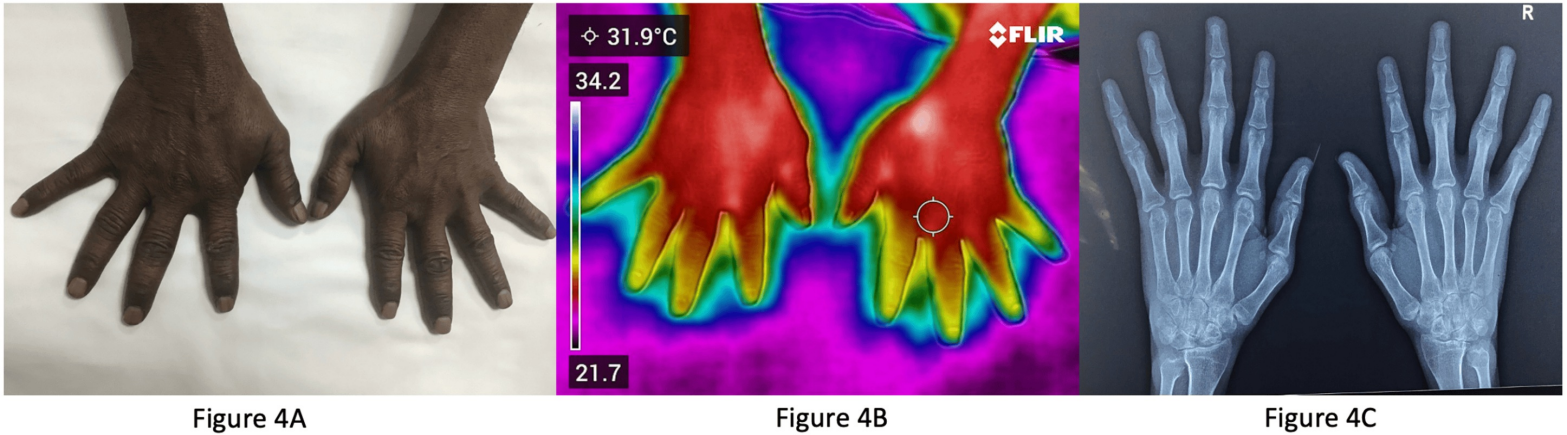
Patient with long-standing rheumatoid arthritis (RA) 4A: Clinical image of another patient with long-standing RA with swelling of the right wrist; 4B: Infrared thermography of the same patient with no increased temperature at the right wrist compared to the other joints; 4C: X-ray wrist of the same patient showing evidence of damage with reduced joint space and erosions.

Figure 4A shows the clinical image of another patient with long-standing RA with evident swelling of the right wrist.

Figure 4B shows IRT of the same patient with no increased temperature at the right wrist compared to the other joints. Figure 4C shows X-ray wrist of the patient with evidence of damage with reduced joint space and erosions. Ultrasonography showed no evidence of active synovitis, indicating that the swelling was due to structural damage rather than ongoing inflammation.

Out of the 50 patients, four patients were in clinical remission and had no active joints clinically. However, IRT detected increased temperature in eight joints.

**Figure 5:**
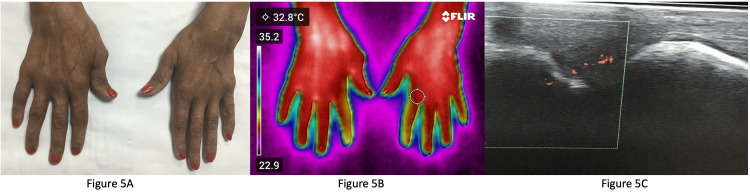
Image of a patient in clinical remission 5A: Clinical image of a patient who was in clinical remission with no active joints clinically; 5B: Infrared thermography of the same patient showing increased temperature at the bilateral wrist and the right first metacarpophalangeal (MCP) joints; 5C: Synovitis with power doppler uptake in the right first MCP joint.

Figure 5A shows the clinical image of a patient who was in clinical remission with no active joints clinically.

Figure 5B shows IRT of the same patient with increased temperature at the bilateral wrist and at the right first MCP joint. Figure 5C shows synovitis with power doppler uptake in the right first MCP joint. These findings suggest that thermography could detect joints with subclinical synovitis. This was confirmed by ultrasonography.

**Figure 6:**
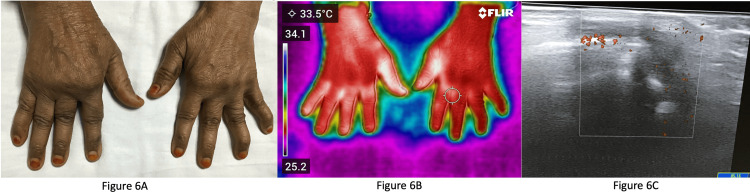
Image of a patient with rheumatoid arthritis (RA) and deformed hands 6A: Clinical image of a patient with deformed RA with volar subluxation at the right metacarpophalangeal (MCP) joint; 6B: Infrared thermography of the same patient showing increased temperature at the right wrist, bilateral first MCP and proximal interphalangeal (PIP), right second, third, and fourth PIP joints; 6C: Ultrasonography showing synovitis with power doppler uptake in the right wrist.

Figure 6A shows a patient with deformed RA with volar subluxation at the right MCP joint.

This patient had a positive bilateral MCP squeeze test, whereas other joints were clinically not active. Her erythrocyte sedimentation rate (ESR) was 36 mm/first hour and she had low disease activity as per the DAS28 score. Figure 6B shows IRT of the same patient, demonstrating increased temperature at the right wrist, bilateral first MCP and PIP joints, and right second, third, and fourth PIP joints (areas in white show increased temperature compared to surrounding red areas which are at normal temperature). Figure 6C confirms synovitis with power doppler uptake in the right wrist. These findings suggest that the patient had active synovitis as per ultrasonography and IRT could detect areas of increased temperatures, though it was clinically not evident.

**Figure 7:**
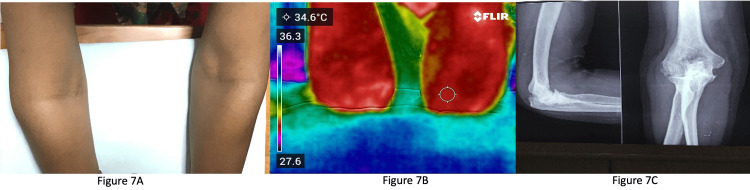
Images of a patient with a swollen right elbow 7A: Clinical image of a patient with a swollen right elbow; 7B: Infrared thermography of the right elbow joint depicting increased temperature; 7C: X-ray of the elbow joint showing reduced joint space with secondary osteoarthritis changes.

Figure 7A shows the clinical image of a patient with a swollen right elbow.

On examination, the patient had tenderness over the right elbow. Figure 7B shows IRT of the right elbow joint depicting increased temperature. An X-ray of the elbow joint (Figure 7C) showed reduced joint space with secondary osteoarthritic changes. Hence, it was difficult to interpret whether this joint was active or damaged. Ultrasonography of the right elbow joint showed erosion with active synovitis, hence the clinical findings were attributed to both activity and damage.

**Figure 8:**
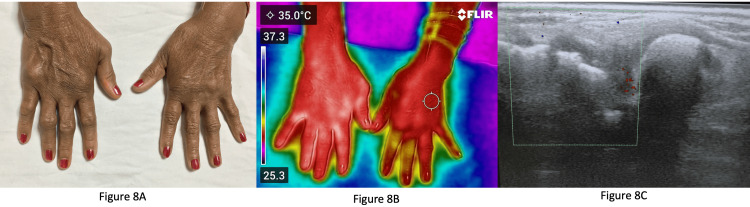
Image of a patient with deforming disease 8A: Clinical image of a patient with deforming disease; 8B: Infrared thermography image indicating increased temperature through all the metacarpophalangeal (MCP) and proximal interphalangeal (PIP) joints; 8C: Ultrasonography image of the right fifth MCP and PIP with synovitis and power doppler uptake.

Figure 8A shows the clinical image of a patient with deforming disease with swelling over multiple PIP and MCP joints. 

She had severe pain over the right fifth MCP and PIP, and had flexion deformity at the fourth and fifth PIP joints. Clinically, it was difficult to distinguish activity vs damage. Figure 8B shows the IRT image, indicating increased temperature through all the MCPs and PIPs, reflecting increased inflammation. Figure 8C shows the ultrasonography image of the right fifth MCP and PIP with synovitis and power doppler uptake.

ROC analysis

**Figure 9:**
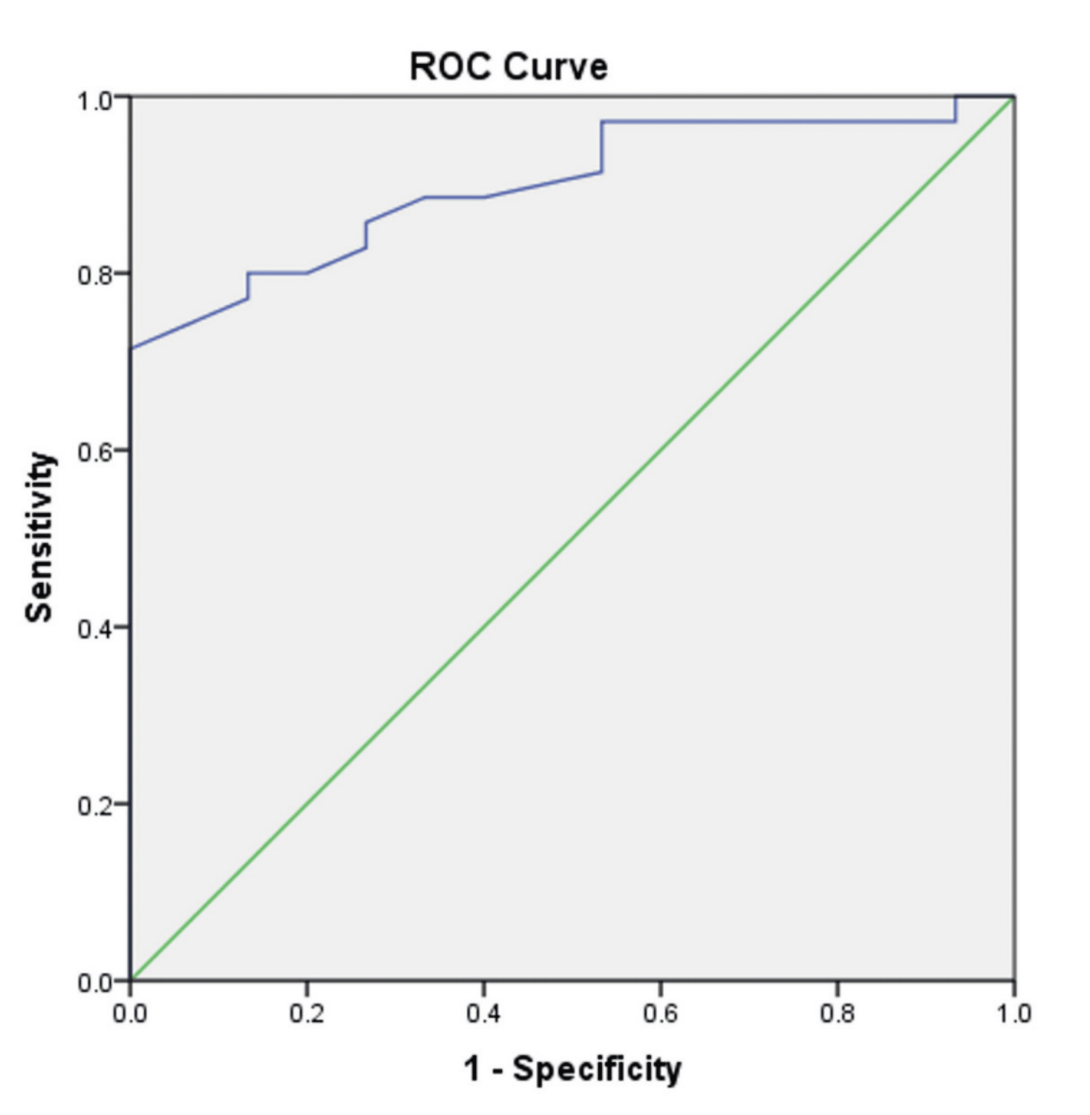
Receiver operating characteristic (ROC) curve for infrared thermography ROC curves detecting moderate/high disease activity (DAS28-ESR >3.2) versus remission/low activity (DAS28-ESR ≤ 3.2) in patients with rheumatoid arthritis; DAS24-ESR: Disease Activity Score in 28 joints using Erythrocyte Sedimentation Rate.

The ROC analysis evaluated IRT’s ability to discriminate between moderate/high disease activity (DAS28-ESR >3.2, n=35) and remission/low activity (DAS28-ESR ≤3.2, n=15). The AUC was 0.84 (95% CI: 0.73-0.95), indicating excellent discriminatory performance. At an optimal T mean cut-off of 33.45°C, IRT achieved a sensitivity of 83% and specificity of 73%, correctly identifying 83% of patients with clinically significant disease activity and 73% of those with remission or low activity (Figure 9).

## Discussion

The current study analyzed 1400 diseased joints and 1400 normal joints, out of which 126 were swollen, and 297 were tender. The mean DAS28-ESR score of the study population was 4.59, with a mean ESR of 44 mm/first hour. Forty-two percent patients had high disease activity, and 28% had moderate disease activity. The T mean in all 28 joints in patients with RA was statistically significant when compared to that of the normal subjects. This result was similar to the study done by Frize et al. [21], where they first proved that thermal imaging could detect inflamed joints in patients with RA; they had taken only 18 normal subjects and 13 patients with RA. They did not mention the disease activity of those patients, and hence, it was difficult to interpret the disease burden taken into consideration. The current study also showed a moderate positive correlation between DAS28 score and the T mean, indicating that thermography could discriminate patients based on the disease severity. The study included 70% patients with moderate and high disease activity; hence, the number of patients with low disease activity was too small (10/50) to arrive at a meaningful significance. But the T mean in patients with low disease activity was higher than in the normal population and slightly higher than in patients under remission.

Tan et al. [26] in 2020 had compared clinical joint assessment, thermography findings, and ultrasonographic parameters (total grey scale score and total power doppler score) and found a good correlation between swollen tender/non-tender joints and higher T mean. The study did not mention the DAS28 score and considered only clinical joint assessment. Moreover, it took into account only wrist joint evaluation. In the current study, we have taken all 28 joints into account and have considered the DAS28-ESR score for disease activity assessment. We performed an ultrasound only in patients who had discordance between their clinical and thermographic parameters.

Our findings also match the study done by Pauk et al., where dynamic IRT correlated with disease activity in patients with RA. They did not take joint temperature, but considered the finger examination protocol, where thermographic details at the static, post-cooling, and post-rewarming phases were considered [27]. Though Pauk et al. used a different thermographic protocol from ours, they demonstrated significant differences in the thermographic patterns in patients with high and moderate disease activity compared to healthy subjects.

Our study also revealed some novel and intriguing findings that have not been previously described in the literature. IRT could pick up tenosynovitis in two patients in whom clinical examination could not differentiate between arthritis and tenosynovitis. IRT in these two patients showed a pattern of increased temperature extending away from the joint, and these findings were confirmed by ultrasonography. Such a specific utility of IRT has not been documented previously, but further studies need to be carried out in larger numbers to validate these findings. But this utility of IRT is a potential area to be explored.

The current study also picked up synovitis in 101 out of 126 swollen joints, which was also confirmed by ultrasonography. Out of the remaining 25 joints, 15 had normal temperature and their X-rays showed evidence of damage. IRT could not detect increased temperature in 10 joints where ultrasonography revealed synovitis. This could possibly be because of the low-resolution camera used in our study. These joints may have been detected if we had used a better camera with higher resolution. This result mirrors the recent study published by Tan et al. where they compared swollen wrist joints with thermography parameters and found a good correlation [28]. They found that swollen joints, regardless of the tenderness status, mirrored the ultrasonography findings. They also found that the high joint temperature detected by thermography in swollen joints corresponded to higher power doppler scores and grey scale score. However the thermal camera used by Tan et al. was a high performance one with better resolution and was almost 10 times costlier than the one used in our study.

Our study detected increased temperature in eight joints in patients on clinical remission, which was confirmed by ultrasonography. Out of the eight, five were MCP and three were wrist joints. This is a very important finding because subclinical synovitis is usually missed. Ultrasonography might not be available in resource-limited settings and expertise is required to detect subclinical synovitis. IRT, being cost effective and easy to handle, could be potentially used to detect subclinical synovitis. This finding needs validation in a large population and needs to be compared with ultrasonography.

IRT could differentiate active joints from damaged joints in 10 selected cases, which were clinically indistinguishable. Seven joints which were labeled as damaged by the treating physician had synovitis detected by ultrasonography. IRT showed increased temperature in all the seven joints, indicating that this could be a potential option to discriminate active joints from damaged ones. This finding also needs validation in a large population and the exact temperature to differentiate activity from damage must be determined. This can be done by using a higher resolution camera for thermal imaging, along with better image processing and data analysis.

The findings of increased temperature detected by IRT in patients with RA when compared to normal subjects mirrors the prior studies [21,27] and opens a new arena for further research in this field. We found that IRT almost mirrors the DAS28-ESR score, which we use clinically to assess disease activity in patients with RA. These findings are encouraging. Researchers can pursue further studies in this field and validate the use of IRT in RA. 

Ivorra et al., in a recent study, developed and validated two novel composite disease activity indices (Thermographic Disease Activity Index (ThermoDAI) and ThermoDAI-­CRP) based on the thermography of hands and machine learning, in order to assess disease activity easily, rapidly, and without formal joint counts [29]. Both indices showed moderate-to-strong correlations with ultrasound-detected synovitis and established clinical indices (Clinical Disease Activity Index (CDAI), Simplified Disease Activity Index (SDAI), DAS28-CRP), outperforming measures of patient self-assessment. This study showed that thermography could be utilized as a fast, easy, and accurate tool to document inflammation without doing a formal joint count. Moreover, it could be incorporated into telemedicine for better remote consultations in patients with RA. However, the camera used by Ivorra et al. was expensive compared to the one we used and the technique used for the analysis of the images was complex, which would be practically difficult to do in our clinical scenario.

Additionally, a recent review on the subject underscored the importance of validating thermography for RA and highlighted the necessity for further research to evaluate the effectiveness of thermal imaging in distinguishing disease activity levels in patients with RA [30]. The current study assessed almost 2800 joints (1400 diseased joints and 1400 normal joints) with thermal cameras. It is the first of its kind to assess disease activity using IRT across all the 28 joints, and using other disease activity measures like the DAS28-ESR score. This study described the normative values of T mean across all the 28 joints in a normal population. Besides assessing disease activity, it also demonstrated IRT's potential utility in detecting tenosynovitis, subclinical synovitis, and in differentiating active joints from damaged ones. Hence further studies are required to explore the utility of IRT in detecting these findings. 

This study is not without its limitations. The sample size was relatively small, which may limit the generalizability of the findings to a broader population. This pilot study did not formally assess inter- or intra-observer variability due to the use of a single trained operator and limited sample size, and hence future studies should include multiple operators and formal variability analyses to confirm thermography’s reproducibility. The device used in the study was of lower resolution and this could have reduced its efficacy in detecting subtle temperature variations. Hence, further studies with a device with better resolution would improve the quality of images. Moreover, the utility of thermal imaging for detecting deeper joints like shoulder and hip joints is challenging and needs further studies. Non-visualization of the joint structures and the inability to perform guided procedures are major drawbacks of IRT. Lastly, we did not perform ultrasonography in all the patients. It was carried out only in patients who had a discordance between clinical and thermography results.

## Conclusions

IRT using a low-cost, compact device demonstrated significantly higher mean joint temperatures in patients with RA compared to healthy controls across all 28 joints, with a moderate positive correlation to DAS28-ESR scores. ROC analysis demonstrated good overall diagnostic performance, with high sensitivity and acceptable specificity at the selected temperature cut-off. These findings support IRT as a promising non-invasive, objective tool for evaluating joint inflammation in RA, particularly in resource-limited settings where advanced imaging like ultrasonography or MRI may not be readily available.

Notably, this pilot study revealed novel applications of IRT, including the detection of tenosynovitis (pattern of temperature extension along tendons), subclinical synovitis in patients in clinical remission, and differentiation between active inflammation and structural joint damage in swollen joints. While the small sample size, use of a lower-resolution camera, and selective ultrasonography limit generalizability, these results are encouraging and warrant validation in larger, multicenter cohorts. If further confirmed, IRT could evolve into a user-friendly, cost-effective adjunct for routine RA monitoring, potentially integrated into mobile platforms for telemedicine applications.
